# Intermittent Fasting on Human Health and Disease

**DOI:** 10.3390/nu15214491

**Published:** 2023-10-24

**Authors:** Denisa Marilena Margină, Cristina Manuela Drăgoi

**Affiliations:** Department of Biochemistry, Faculty of Pharmacy, Carol Davila University of Medicine and Pharmacy, 6 Traian Vuia Street, 020956 Bucharest, Romania

Chronic non-communicable diseases (NCDs) are the leading cause of morbidity and mortality worldwide, but most of all in industrialized countries, and are fundamentally correlated to improper nutrition and impaired lifestyle behaviours [1]. Also, the ageing process, with all its associated chronic disparities, is accelerated by imbalanced nutrition, altered feeding behaviour and lifestyle in general, benefiting from changes in these factors [2,3]. The brain cellular network is highly dependent on the bioenergetics homeostasis, as it is negatively influenced by sedentary and imbalanced food intake; under such conditions, decreased neuroplasticity and impaired calcium homeostasis are reported, changes that make the subjects more susceptible to neurodegeneration and stroke [4].

Over-nutrition is an important risk factor for various human diseases, including neurodegenerative diseases, metabolic disorders, and cancers; diet, one of the most important lifestyle factors, is strongly correlated either with a good health status or with the development of many chronic conditions such as obesity, cardiovascular disease, hypertension, stroke, type 2 diabetes, metabolic syndrome, some cancers, and some neurological diseases [5,6,7]. In this context, changes in dietary patterns are highly discussed in recent research in the quest for discovering some kind of *magic bullet* against all the above-mentioned health impairment issues which represent a heavy burden on the quality of life all over the world [3,4,6,7,8,9,10]. In the framework of changes in dietary patterns, there are two main approaches: either a reductive shift of the caloric intake/change in food quality or an important modification of feeding behaviour in regard to the timeline of eating. Regarding food composition, research shows that enriching human diet in polyunsaturated fatty acids as well as natural antioxidants leads to an improvement of cardio-metabolic risk [3,6,11,12,13,14,15,16,17,18,19].

The second approach is represented by intermittent fasting, comprising eating programs of individuals that undergo long time periods (16–48 h) with very reduced/no energy/food intake, alternating with periods of normal food consumption; under the “umbrella” of intermittent fasting, several forms are encountered—temporary food avoidance or time-restricted feeding or other similar fasting patterns; studies, either on animal models or human ones (most of which include the Ramadan model) show that intermittent fasting improves the metabolic outcome, diminish inflammation, improve weight management, increase insulin sensitivity and even counteract ageing in both normal-weight and overweight subjects [2,4,5,8,18,19,20,21,22,23,24,25].

Studies show that this kind of approach has beneficial metabolic effects, improving mitochondrial function and stimulating fatty acid oxidation as well as increased clearance of cellular lipids, regulating glucose homeostasis, activating adaptive cellular stress responses, reducing oxidative stress and diminishing inflammatory responses, inducing DNA repair and autophagy, etc. [5,6,7,18,26,27]. Intermittent fasting regimens are able to regulate metabolic routes by influencing the target of rapamycin (TOR) pathway and circadian clock, profoundly changing the gut microbiota composition, influencing the sleep quality and other lifestyle factors [10,28].

All these acute changes in cellular and metabolic pathways are associated with a decrease in chronic disease burden as well as the increase in protective molecule levels [12].

Intermittent fasting has been shown to have important health benefits in the prevention and management of chronic diseases and in the process of biological aging. The effect of intermittent fasting on hormonal circadian rhythms has also received particular attention, mainly due to the tremendous importance of circadian hormonal impact on normal physiology and its pathophysiological involvement in clinical endocrinology [10,21,22,29,30]. However, more profound research is needed to determine the efficacy and safety of intermittent fasting in humans, and to fully understand the underlying mechanisms by which this nutritional approach exerts its effects in human health and disease.

In this context, the current Special Issue “Intermittent Fasting on Human Health and Disease” is aiming to gather current knowledge in the field and to update the information regarding the clinical relevance of this dietary pattern, to further collect arguments in support of already available data regarding the potential of intermittent fasting regimens to be efficacious and offer a certain non-pharmacological approach to improving health at the population level, and induce public health benefits.

This Special Issue welcomes contributions from researchers and nutrition experts in all related fields; specialists are invited to submit their original work, review articles and communications, clinical, preclinical and experimental studies that are linked to the interplay between nutrition, all forms of restricted eating and their effects on chronic disease.

These findings will support health professionals and nutritional practitioners in the shaping of new ideas and coach their patients to embrace improved eating behaviours.
